# Complete Genome Sequence of Cupriavidus necator KK10, an Azaarene-Degrading and Polyhydroxyalkanoate-Producing Soil Bacterium

**DOI:** 10.1128/MRA.00423-21

**Published:** 2021-07-15

**Authors:** Jiro F. Mori, Miho Nagai, Robert A. Kanaly

**Affiliations:** a Department of Life and Environmental System Science, Graduate School of Nanobiosciences, Yokohama City University, Kanagawa, Yokohama, Japan; Indiana University, Bloomington

## Abstract

Cupriavidus necator KK10 has been investigated in azaarene and diesel fuel biodegradation studies and is capable of polyhydroxyalkanoate (PHA) production. Its complete genome sequence revealed two closed circular sequences, the chromosome (6.68 Mb) and plasmid (1.67 Mb). The KK10 genome carries functional genes potentially responsible for azaarene biodegradation and polyhydroxyalkanoate biosynthesis.

## ANNOUNCEMENT

Cupriavidus necator KK10, a member of the *Betaproteobacteria*, was isolated from a cattle pasture soil bacterial consortium by streaking on Noble agar with the heavy fraction of a diesel fuel as the carbon source (1–5). KK10 biotranforms azaarenes and single-ring aromatic compounds (4–6) and produces polyhydroxyalkanoates (PHA). Sequencing the genome of KK10 expands our understanding of this versatile and potentially useful bacterium.

Hybrid assembly was conducted by utilizing short-read (DNBSEQ-G400; MGI Tech, China) and long-read (GridION X5; Oxford Nanopore Technologies, UK) sequencing to close the chromosomal and plasmid sequences similarly to a method described previously (7). The strain was grown on 20 mM glycerol in Stanier’s basal medium for 3 days at 30°C by rotary shaking (150 rpm in darkness), DNA was extracted with the NucleoBond high-molecular-weight (HMW) DNA kit (Macherey-Nagel, Germany), and a DNBseq library was prepared with the MGIEasy FS DNA library prep set (MGI Tech). A total of 6,210,182 reads were obtained with 200-bp paired-end read lengths that were trimmed and quality filtered using Cutadapt v. 2.7, SeqKit v. 0.11.0, and Sickle v. 1.33. Then, 1,000 ng of genomic DNA was barcoded using native barcoding expansion for GridION analysis, and a library was created using a ligation sequencing kit (SQK-LSK109; Oxford Nanopore Technologies; after adapter ligation, >3-kb fragments were enriched). From the GridION platform, using R9.4.1 flow cells and Guppy v. 4.0.11 for live base-calling, a total of 130,465 reads with an average length of 6,801 bp were generated. Raw reads were trimmed and quality filtered using Porechop v. 0.2.3 and Filtlong v. 0.2.0 (minimum length, 1,000 bp). All software was used with default settings unless otherwise indicated. *De novo* hybrid assembly was conducted after trimming and quality filtering with Unicycler v. 0.4.7 (8) and was validated with Bandage v. 0.8.1.

The complete genome was determined (8,350,386 bp; 255× coverage) and consisted of a circular chromosome (6,679,877 bp) and plasmid (1,670,509 bp) with an overall G+C content of 65.6%. The NCBI Prokaryotic Genome Annotation Pipeline v. 5.1 was utilized to perform gene annotation, whereby 7,750 coding sequences (CDSs), 15 rRNAs, 65 tRNAs, and 2 transfer-messenger RNAs (tmRNAs) were identified; 6,142 (79.25%) and 1,608 (20.75%) CDSs were located on the chromosome and plasmid, respectively.

According to homologous gene identification using the Integrated Microbial Genome and Microbiomes (IMG/MER) system, a *phaCABR* gene cluster, which includes genes responsible for PHA biosynthesis (9) (PHA polymerase, beta-ketothiolase, acetoacetyl coenzyme A reductase, and PHA synthesis repressor, respectively) was found located on the chromosome and supported PHA synthesis in KK10. At the same time, homologues of the *indAB* genes, which encode an indole monooxygenase (10), were found on the main chromosome, and their identification confirmed the capabilities of KK10 to biotransfrom azaarenes such as indole to produce indigoids (4, 5).

### Data availability.

The sequences were deposited in NCBI GenBank under the accession numbers CP073677 and CP073678 and in the IMG/MER database under accession number 2913661577. The raw sequences are available from SRA accession numbers SRR14308055 and SRR14308056 under BioProject number PRJNA722091 and BioSample number SAMN18744514.
